# A systematic literature review on sustainability issues along the value chain in insurance companies and pension funds

**DOI:** 10.1007/s13385-023-00349-1

**Published:** 2023-05-10

**Authors:** Laura Iveth Aburto Barrera, Joël Wagner

**Affiliations:** 1 Department of Actuarial Science, Faculty of Business and Economics, University of Lausanne, Lausanne, Switzerland; 2 Swiss Finance Institute, University of Lausanne, Lausanne, Switzerland

**Keywords:** Sustainability, ESG, Insurance companies, Pension funds, Value chain

## Abstract

Sustainability is now a priority issue that governments, businesses and society in general must address in the short term. In their role as major global institutional investors and risk managers, insurance companies and pension funds are strategic players in building socio-economic and sustainable development. To gain a comprehensive understanding of the current state of action and research on environmental, social and governance (ESG) factors in the insurance and pension sectors, we conduct a systematic literature review. We rely on the PRISMA protocol and analyze 1 731 academic publications available in the Web of Science database up to the year 2022 and refer to 23 studies outside of scientific research retrieved from the websites of key international and European organizations. To study the corpus of literature, we introduce a classification framework along the insurance value chain including external stakeholders. The main findings reveal that risk, underwriting and investment management are the most researched areas among the nine categories considered in our framework, while claims management and sales tend to be neglected. Regarding ESG factors, climate change, as part of the environmental factor, has received the most attention in the literature. After reviewing the literature, we summarize the main sustainability issues and potential related actions. Given the current nature of the sustainability challenges for the insurance sector, this literature review is relevant to academics and practitioners alike.

## Introduction

Climate change, social inequality, and corporate governance are some of the biggest challenges facing society today. These concerns are part of what is known as the environmental, social and governance (ESG) factors, which shed light on the obligations of companies and governments to the community in building a sustainable economy. In addition, and as original motivation, ESG factors are important to insurance companies and pension funds when assessing the associated financial risks and opportunities in their firms. As an illustration of the alarming state on the environmental front, the latest report from the Intergovernmental Panel on Climate Change (IPCC, see [67]) has revealed that “there is at least a 50 percent probability that global warming will reach or exceed 1.5 $^{\circ }$C in the short term, even under the very low greenhouse gas emissions scenario, requiring immediate action to combat climate change.” Although it is difficult to develop detailed scenarios and models, the insurance industry can easily envision the impact that natural disasters will have on their risk management and underwriting in the future. Similarly, social and governance concerns have also been thrust into the spotlight by spectacular scandals in many industries, to a point that, as defined by the UN Environment Programme (UNEP), insurance companies and pension funds play a key role in acting on ESG issues [86].

Based on the extant academic literature and recent publications outside of scientific research, the objective of this work is to provide a comprehensive understanding of what is currently being done on sustainability in the insurance and pension sectors. To this end, we analyze 1 731 academic publications available through the Web of Science database using the PRISMA protocol for systematic literature review. We supplement this corpus with 23 publications retrieved from (insurance) organizations’ websites. We introduce a framework guided by the Principles for Sustainable Insurance (PSI) and the value chain of insurance companies (primary activities), insurance strategy (supporting activity), and external stakeholders and reporting to classify the literature. Our main results include a current review of the state of research and an overview on the sustainability issues and related actions along the categories of our framework.

Perceptions on sustainability have evolved over the past decade. When the topic of sustainable development emerged in the 1970s, it focused primarily on environmental issues. The first world conference on the environment was held in Stockholm in 1972 and established the UNEP as a global agency to manage the environmental agenda [94]. Many conferences later, an important initiative was launched by the UN, the so-called Millennium Development Goals [93] in 2000, encompassing eight major goals related to social and environmental issues to be achieved by 2015. However, practical standards and guidelines have only been developed since 2015. Two landmark events demonstrated the importance of finding appropriate solutions to current global challenges in 2015. These are the Paris agreement on Climate and the UN Sustainable Development Goals (SDGs, see [46]). While the first aims to limit global warming to 1.5 $^{\circ }$C [20], the SDGs have at their core 17 goals that members are expected to achieve by 2030. They address, social and environmental issues such as, no poverty, zero hunger, clean water and sanitation, responsible consumption and production, and climate action, among others. While until 2000, there were few initiatives and standards to assess sustainability challenges, we observe that in recent years, and especially since 2015, there has been an increasing number of developments committed to sustainable development.

In recent years, the need for addressing ESG challenges has increased dramatically. Although insurance companies and pension funds are not directly linked to any of the sustainability dimensions, awareness has changed recently. The insurance business processes including risk and investment management are strongly linked to ESG factors, and many companies have embarked on a journey for sustainability adaptation. In the present study we review the existing academic publications and study the research concerned with sustainability in the insurance sector. We find that the environmental aspect has received the most attention, especially in the underwriting and investment areas, which underlines that climate change is a key concern of the industry. On the other hand, we find that areas related to insurers’ activities along claims processing are less researched in academia, while the area of sales and marketing receives the lowest attention outside the academic world. Overall, the main issues that we identify include a lack of appropriate integration of sustainability in the strategy and operations as well as the absence of standardized quantitative indicators. The actions proposed in the literature indicate embedding ESG issues into all processes.

This work is organized as follows: in Sect. 2, we lay out the relationship between insurance and sustainability and discuss the challenges the sector faces in addressing sustainability issues. In Sect. 3, we discuss the literature review methodology, statistics on the corpus of literature, and introduce our classification framework along the insurance value chain. In Sect. 4, we review key aspects of the retrieved literature based on the proposed classification framework and discus the main sustainability issues and potential related actions. We conclude in Sect. 5.

## Sustainability in insurance

Insurance is an essential pillar of global economic activity. Through their investments, but also given their exposure to losses and claims payments as well as in their role as risk managers, insurance companies and pension funds are concerned by the ESG factors. Therefore, it is important to understand and develop viable solutions to cope with the sustainability challenges but also to take advantage of market opportunities [47]. In this section, we describe the relationship between industry and sustainability, discuss the current state of the sector and key challenges, and provide selected insights into current practices in the area of sustainability.

ESG factors have established as a standard for describing sustainability issues and as target challenges when paving the way for achieving higher levels of sustainability in financial markets. As investors and risk managers, insurers and pension funds play a key role in ensuring sustainable development [6]. Insurers are exposed to sustainability issues on both underwriting and investment sides. Pricing, underwriting and claims management activities are concerned by increasing risks, for example from natural catastrophes. Given the size and duration of their institutional investment portfolios, insurers and pension funds are at the forefront of responsible investment.

In this context, it is important to take a closer look at the individual ESG factors. Concerning the environmental factor (E), we distinguish the effects associated to the two sides of the balance sheet, assets and liabilities. On the liabilities side, insurance is a leading sector in climate change adaptation [43]. Indeed, the sector provides financial resilience to extreme natural events and expertise for risk assessment [32]. For example, insurance companies have already developed products and services that help to reduce greenhouse gas emissions. These products and services include risk transfer solutions for weather-related risks, crop insurance, microinsurance for small farmers, and renewable energy products [32]. On the assets side of the balance sheet, beyond the mere environmentally friendly investing, insurance-linked securities have gained notoriety as protection against insured losses (cf. catastrophe bonds, [59]).

Regarding the social (S) factor, insurers and pension funds are particularly concerned. On the one hand, they must care about workers’ pensions and therefore include workers’ rights, social inclusion, gender equality, child labor, and other SDGs. On the other hand, as principal asset owners, they should invest responsibly and “green”. One of the actions that have been carried out in this field are the microinsurance solutions to fight poverty in low-income countries [50]. In addition, the consideration of sustainable investments by pension funds has increased [1]. Finally, governance (G) is a key factor not only for the insurance sector, but also for all other sectors, since it does not differ significantly from company to company, especially in the financial sector. A better management strategy for a resilient and sustainable business requires a comprehensive approach in which the relationships with stakeholders, in particular customers, governments, and regulators, and all business activities in the value chain are organized and managed in a responsible manner [86]. Therefore, factors such as board diversity, corruption, and bribery, and improving internal controls and risk management are some themes that the insurance industry is already aware of an implementing [29].

Today, the growing trend towards addressing sustainability issues in insurance is unmistakable, as many companies and governments are promoting sustainable development as a major issue worldwide. Since 2000, there have been number of initiatives addressing sustainable development, and highlighting the growing role of insurance in this area [6]. One of the key global initiatives developed explicitly for the insurance industry is the PSI published by the UNEP Finance Initiative [86]. The main objective of the principles is to take a strategic approach to conducting the insurer’s key activities in a responsible manner, and to manage and assess risks and opportunities related to ESG issues in order to be a sustainable company. Currently, several global and national organizations are advocating for sustainability issues. Standards and principles have been developed for the insurance sector for both the asset and liability sides of the balance sheet.

However, despite the importance placed on sustainability globally and the increasing development of initiatives and frameworks for managing ESG issues, there are some challenges raised by practitioners and academics. First, one of the biggest challenges is the access to reliable data. For example, in many regions there is limited access to hazard and exposure data to assess physical climate risks [37]. Another major challenge is the need for a regulatory framework for financial markets. Due to unclear and fragmented regulations, it is difficult for primary insurers to access reinsurance and for investors to evaluate opportunities [32]. Furthermore, regarding environmental issues, specific challenges relate to the liability side of the balance sheet, for example, modeling of various adverse scenarios, sustainable reinsurance structures, and development of climate-related mortality tables [64]. In lower income countries, these challenges are complemented by a lack of insurance awareness, limited acceptance of natural catastrophe insurance, and the absence of domestic insurance [32]. On the asset side, challenges include the need for more green bonds and issuers, the development of new green financial instruments, the need for policy incentives to encourage green investment on a large scale, the failure to price carbon and natural resources, and the need for better climate risk reporting standards [32]. Insurers are increasingly recognizing environmental issues as part of their enterprise risk management [54]. More recently, events like the Covid-19 pandemic and social movements, such as the #MeToo movement against sexual harassment in 2017 and the #BlackLivesMatter movement that resurged in 2020 after George Floyd’s murder, have exposed social failures, and shed light on the poor social practices of some companies and governments, forcing them to initiate change. More specifically, in terms of governance, major challenges are, for example, the diversity of board members and workforce. Diversity and inclusion are important sustainability issues, but they also present opportunities to enhance the reputation and strengthen relationships with employees [29]. Finally, the unclear general definitions of sustainability in the insurance sector also pose risks for defining management roles and responsibilities [29]. Given the multiple facets of sustainability issues, we systemically review the academic literature in the sequel to derive a more comprehensive picture on the issues and related actions.

## Literature review: methodology and statistics

In this section, we first present the search strategy and collection of publications leading to the corpus of selected academic papers and studies outside of scientific research that we call “practitioner” publications. We lay out the inclusion and exclusion criteria used in our review protocol (see Sect. 3.1). Then, we describe the overall statistics on the corpus of academic literature in Sect. 3.2. In Sect. 3.3, we propose a framework to classify the publications along the insurance value chain and external stakeholders. We present in Sect. 3.4 statistics on the academic publications based on the classification introduced in Sect. 3.3. Finally, we present the statistics on the practitioner publications in Sect. 3.5.

### Review strategy and data collection

**Fig. 1:**
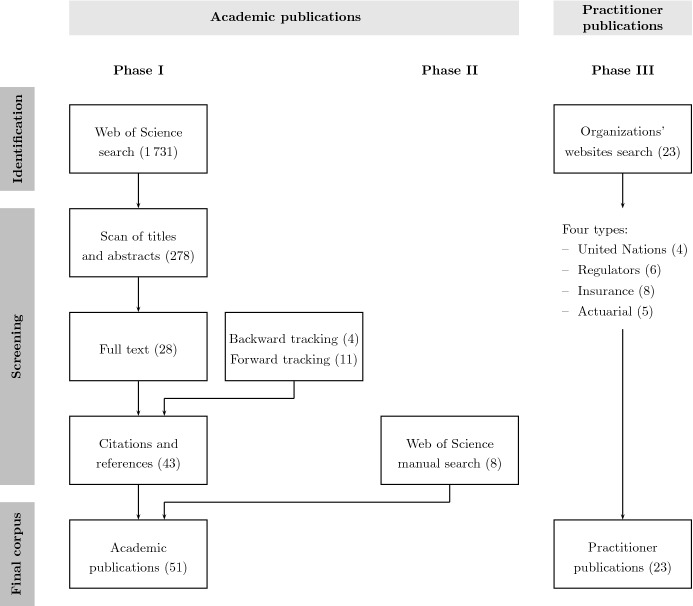
Flow diagram for the identification and screening of records along PRISMA guidelines

We have conducted a literature review to identify and classify existing academic research on sustainability in insurance. For the review, we follow the Preferred Reporting Items for Systematic Reviews and Meta-Analyses protocol (PRISMA, see [62]). Our review is based on a search of the Web of Science Core Collection database for academic publications and of relevant organizations’ websites for practitioner publications. We proceeded in three phases by first running a general query for academic publications, then a complementary manual search, and finally a search for practitioner publications. A flowchart and synopsis of the three phases of our review protocol is presented in Fig. 1.

The PRISMA review protocol used in the first phase consists of three key steps for the first phase. In the first step (identification), we consider all database records and restrict our search by using filters and keywords. Our query process included all years through December 2022 and academic publications recorded by Web of Science. We included English language documents and limited the keywords search to the abstract. The reason for using selected keywords is to consider the concept of sustainability, which refers specifically to insurance and relates to the ESG factors. For the selection, we used the keywords “insur*”, “pension*”, “actuar*”, “sustainab*”, “esg”, “environment*”, “soci*”, “govern*”, and “climate change” in the search string, where the asterisk (*) is a placeholder for any number of other characters. The keywords “insur*”, “pension*”, and “actuar*” make us include all insurance, pension, or actuarial science related publications. The terms “sustainab*” and “esg” have been added to filter for sustainability topics, while the words “environment*”, “soci*”, “govern*”, and “climate change” more precisely relate to the environmental, social and governance topics.^1^ Our search retrieves a total of 1 731 publications.

In the second step, we scan the resulting records and exclude records in specific fields of research that do not relate to our topic (e.g., health fields like emergency medicine, rheumatology, obstetrics gynecology, geriatrics gerontology, surgery, and tropical medicine, and other fields like religion, philosophy, zoology, and government law). We review the titles and abstracts of the remaining records, and exclude 1 453 articles that do not relate to the insurance industry and sustainability issues. After this step, we remain with a total of 278 results. In a third step, we perform a screening of the full texts, which yields 28 publications. The inclusion criteria relate to articles in the insurance industry as a main player in sustainability, i.e., publications should address questions at the crossroads of insurance and sustainable business or ESG factors. We then conduct a forward and backward^2^ literature search for the citing and cited references related to the 28 records and select a total of four and 11 articles, respectively. This leads us to 43 records. Finally, we manually conducted a second phase of search, capturing any insurance-related publications that were not included in the first phase due to the specificity of keywords. A total of eight publications have been added to our final corpus, resulting in a total number of 51 academic publications. In the third phase, we consider studies from relevant organizations from the last three years (2020–2022) and exclude short summaries that are less than five pages long. For our search, we consider the most relevant international and European organizations working in the field of sustainability in insurance. These organizations include programs of the United Nations, standard setters, regulatory authorities, insurance think tanks, insurance companies, and actuarial associations. We categorize the publications into four distinct types according to the organizations’ characteristics, i.e., “United Nations”, “Regulators”, “Insurance” and “Actuarial”. In the United Nations group we retain four publications, in the regulators type we select a total of six records, in the insurance group we select eight publications, and in the actuarial group we consider five records. This gives a total number of 23 practitioner studies. We present more details on the origins of the practitioner studies in Sect. 3.5.

### Statistics on the corpus of academic literature

The 51 publications included in the final corpus stem from 28 journals. The *Geneva Papers on Risk and Insurance—Issues and Practice* (14), *Sustainability* (3), and *Business Strategy and the Environment, Corporate Social Responsibility and Environmental Management, Journal of Business Ethics, Journal of Cleaner Production, Natural Hazards* and *Science* (2), are the journals with the highest number of articles that have published research on sustainability in insurance through 2022 (see Table 1). Of the 14 articles in the *Geneva Papers on Risk and Insurance—Issues and Practice*, 10 studies address climate change, and four articles treat sustainability issues in general. The authors appearing most frequently are Mills (single author of [52–55]) and Johannsdottir (author and coauthor of [42–45]) with four appearances each, all focusing on climate change and environmental sustainability. We note that among the 51 articles in our final corpus, 43 records focus on insurance companies, while the other eight articles discuss pension fund matters.

**Table 1:** Journals with the highest number of publications in the final corpus

Journal	Number of records
Geneva Papers on Risk and Insurance—Issues and Practice	14
Sustainability	3
Business Strategy and the Environment	2
Corporate Social Responsibility and Environmental Management	2
Journal of Business Ethics	2
Journal of Cleaner Production	2
Natural Hazards	2
Science	2

Only the first eight journals are listed. The remaining have one publication each. The journals are ranked by number of records and listed in alphabetical order if equally ranked

**Fig. 2:**
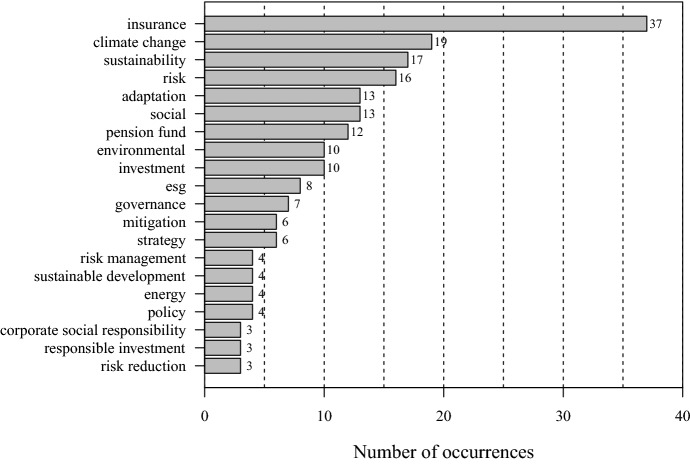
Number of occurrences of the 20 most frequent topics in the keywords. Note: The topic “insurance” includes the keywords, insurance, insure, insurer and insurers; “environmental” term includes environmental, environment and green; “investment” includes investing, investment and invest; “sustainability” includes sustainability, sustain and sustainable

To identify and analyze the most relevant topics, we have examined the frequency of keywords in the corpus overarching the years from 2003 (oldest publication) to end of 2022. In a first step, we report the most frequent topics based on the author’s keywords field in the 51 publications. To form the topics, we have clustered keywords with similar or related meanings. For example, the topic “insurance” includes the keywords insurance, insurer, insure, and insurers; “environmental” includes the keywords of environment, green, and environmental. The term “investment” refers to investing, investment, and invest; “sustainability” includes sustain, sustainability, and sustainable words. Among the six most frequent topics, we find that “insurance” ranks first with 37 occurrences. The topic “climate change” ranks second with 19 occurrences. The topics “sustainability”, and “risk” rank third and fourth with 17 and 16 repetitions respectively. The topics “adaptation” and “social” rank fifth and sixth with 13 records each. The frequency analysis of the 20 most frequent topics that we report in Fig. 2 provides insight into what has been of most interest to research over the past two decades. Besides “insurance”, the keywords climate change, sustainability, social and environmental appear most frequently, which was to be expected given the search query for the selection of records. In order of appearance, climate change issues come first, followed by social and governance issues. We also observe that many keywords are related to the main characteristics of insurance such as risk, mitigation, and strategy.

**Fig. 3:**
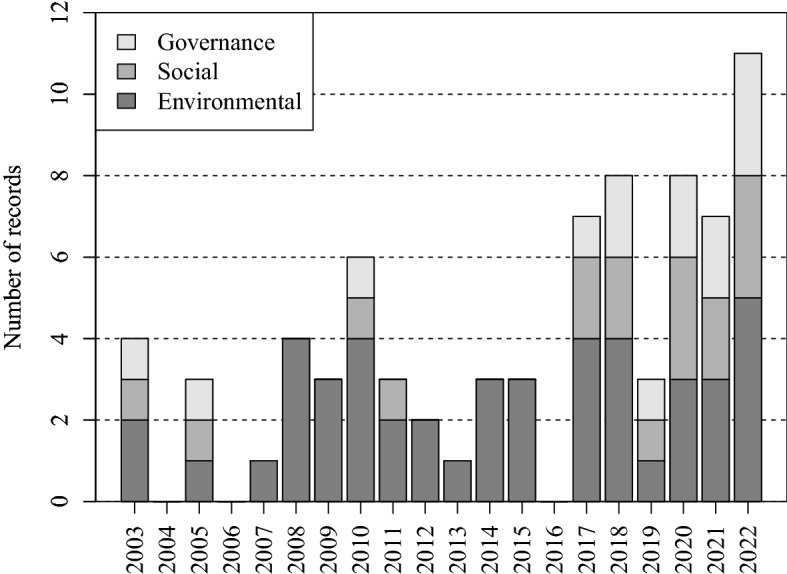
Number of academic publications per ESG factor and per year. Note: A publication can be counted several times if related to several ESG factors

In a second step, we have linked the topic of each academic publication to the three ESG factors: the environmental factor includes topics related to climate change such as natural disasters, pollution, and natural resources; the social factor considers topics related to human rights, gender equality, labor standards, and other issues affecting society; the governance factor includes topics related to corporate governance, board composition, corruption, and bribery. The (general) keywords “ESG” and “sustainable development” are assigned to all three categories. In Fig. 3, we present the resulting number of records by ESG factor and by year. On the first hand, we observe occurrences of publications regarding the environmental factor in almost all years. Moreover, the number of records seems to be increasing over the (most recent) years, from two records in 2003 to five records in 2022. On the second hand, regarding the governance and social factors, most records are found between 2017 and 2022 (although there are also some records in 2005 and 2010). We observe that a certain number of publications appeared in the years after 2007 relating to the publication of the climate change synthesis report by the IPCC [41]. Further, the increase of the number of records from 2017 onwards can be linked to the rising awareness in the scientific community after the Paris agreement [20] and the development of the UN SDGs in 2015 [46]. In addition, we note that all records are either devoted exclusively to the environmental factor or address the three, environmental, social, and governance, factors together. Thus, social and governance factors have never been treated alone in academic publications.

Overall, we observe that there has been a lot of interest in researching environmental issues. This is primarily due to the insurance industry’s concern about the increase in natural disasters and associated risks. Furthermore, climate change is currently given great importance at the international level, and both public and private organizations are actively involved in promoting awareness of environmental issues. On the other hand, ESG practices have come to the forefront in recent years and have become a standard for achieving sustainability in financial markets. For example, ESG investing aims to meet the needs of investors and the public by incorporating long-term financial risks into investments decisions [11]. Therefore, an undeniable increasing trend for research on the insurance industry sustainability issues can be expected in the coming years.

At this stage, the statistics on the collected literature have helped us to get a first impression on the topics that got the most attention from academic research. In the following, we propose a classification framework along the insurance value chain to explore in greater detail the focus of the publications in our corpus.

### Classification along the insurance value chain

To study the research areas covered by the retrieved corpus of literature and to more systematically assess extant research and potential gaps, we introduce a classification of key insurance sector activities guided by the PSI [86] and using previous works linking an insurer’s value chain processes to sustainable development (see, e.g., [45, 81]). The first principle of the PSI encompasses the strategy and the operations of an insurance company. Principles 2 and 3 address external stakeholders of the insurance industry, namely, clients, suppliers, investors, governments and regulators. Furthermore, the fourth principle relates to the accountability and reporting of insurers. We distinguish supporting activities, primary activities, and external stakeholders and reporting. We provide further background information on the nine categories when discussing the literature in Sect. 4.1.

**Fig. 4:**
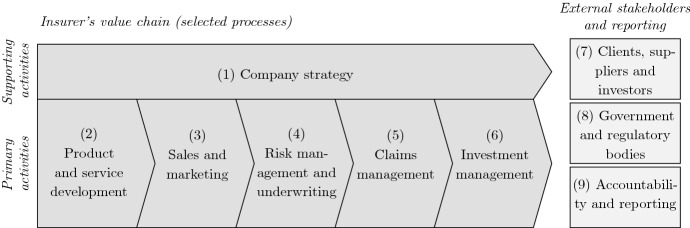
Classification framework with nine categories along the insurance value chain and stakeholders

As illustrated in Fig. 4, we consider a framework based on nine main categories, including the value chain and relevant externalities. As a key representative for supporting activities in an insurance company we consider the company strategy (1). In the primary activities (operations), we consider product and service development (2), sales and marketing (3), risk management and underwriting (4), claims management (5), and investment management (6). Insurance companies are liable to several external stakeholders including clients, suppliers and investors (7) and the government and regulatory bodies (8) linked to their accountability and reporting (9). The proposed framework allows us to review which insurance activities are more researched (and concerned) with ESG issues (see Sect. 3.4 and Table 2).

### Statistics on the academic publications along the value chain categories

In Table 2 we report the number of publications of the final corpus that we have classified in each of the nine categories introduced in Sect. 3.3 (see Fig. 4). Thereby a publication may refer to one or more categories. Additionally, in each category we consider the three ESG factors to quantify the number of records relating to each factor (E, S, or G). This split provides insights on which share of the extant literature covers these specific topics.

**Table 2:** Number of academic publications per category and ESG factor

	Number of records			
Category	Overall	E	S	G
(1) Company strategy	16	13	5	5
(2) Product and service development	14	14	3	2
(3) Sales and marketing	3	2	1	0
(4) Risk management and underwriting	27	26	5	4
(5) Claims management	2	2	0	0
(6) Investment management	22	18	10	9
(7) Clients, suppliers and investors	6	3	4	1
(8) Government and regulatory bodies	20	19	5	5
(9) Accountability and reporting	5	3	4	3

The column “Overall” refers to the total number of academic publications in a given category. The columns “E”, “S” and “G” stand for the environmental, social and governance factors. A publication may relate to several categories and a category to several ESG factors

The categories receiving the highest attention from academic research include the risk management and underwriting (4), the investment management (6) and the government and regulatory bodies (8). In each of these categories, we record over 20 publications, with most of them related to environmental issues. The categories company strategy (1) and product and service development (2) rank fourth and fifth in terms of number of publications. All the other activities receive much less attention, in particular sales and marketing (3) and claims management (2) with merely three and two records, respectively. We observe that in most categories academic research focuses primarily on environmental factors (E). In Table 3 we summarize the academic publications included in each category. More details and a complete classification of the 51 academic publications in the final corpus are provided in Table 7 in the Appendix. The summary table includes information on the regions where the study was conducted, the research method used, and the key contents and main results for each publication. Furthermore, for each publication we indicate the relevant categories (1 to 9) and ESG factors to which it refers.

**Table 3:** Classification of the academic references (in alphabetical order) along the value chain categories

Category	References
(1) Company strategy	Alda [1]; Altarhouni et al. [3]; Begum et al. [9]; Ho et al. [38]; Johannsdottir [42]; Johannsdottir et al. [45]; Johannsdottir and McInerney [44]; Linnerooth-Bayer et al. [50]; Mills [54]; Pierro and Desai [66]; Scholtens [76]; Sethi [77]; Sievänen et al. [78]; Stricker et al. [81]; Thirawat et al. [85]; Woods and Urwin [99]
(2) Product and service development	Begum et al. [9]; Dahlström et al. [19]; Johannsdottir et al. [43]; Johannsdottir et al.[45]; Keskitalo et al. [48]; Mills [52]; Mills [54]; Nogueira et al. [58]; Qing and Liang [69]; Rempel and Gupta [70]; Stahel [79]; Stricker et al. [81]; Thirawat et al. [85]; Wilkins [97]
(3) Sales and marketing	Lee et al. [49]; Stricker et al. [81]; Ward et al. [95]
(4) Risk management and underwriting	Ball et al. [7]; Begum et al. [9]; Botzen et al. [12]; Dahlström et al. [19]; Dlugolecki [21]; Garayeta et al. [27]; Glaas et al. [30]; Herweijer et al. [37]; Johannsdottir et al. [43]; Linnerooth-Bayer et al. [50]; Mills [52]; Mills [53]; Mills [54]; Mills [55]; Müller-Fürstenberger and Schumacher [56]; Nogueira et al.[58]; Pagano et al. [61]; Paudel [63]; Phelan [65]; Qing and Liang [69]; Sato and Seki [72]; Schiller and Crugnola-Humbert [75]; Sethi [77]; Stahel [79]; Stricker et al. [81]; Thirawat et al. [85]; Wilkins [97]
(5) Claims management	Sato and Seki [72]; Stricker et al. [81]
(6) Investment management	Alda [1]; Alda [2]; Autenne et al. [5]; Begum et al. [9]; Chiaramonte et al. [15]; Dlugolecki [21]; Gatzert and Reichel [28]; Herweijer et al. [37]; Johannsdottir et al. [45]; Lee et al. [49]; Mills [52]; Mills [54]; Owadally et al. [60]; Qing and Liang [69]; Rempel and Gupta [70]; Risi [71]; Sethi [77]; Sievänen et al. [78]; Stahel [79]; Stechemesser et al. [80]; Thirawat et al. [85]; Woods and Urwin [99]
(7) Clients, suppliers and investors	Lee et al. [49]; Mills [52]; Mills [54]; Risi [71]; Sethi [77]; Wilkins [97]
(8) Government and regulatory bodies	Ball et al. [7]; Benali and Feki [10]; Brogi et al. [14]; Chiaramonte et al. [15]; Dahlström et al. [19]; Garayeta et al. [27]; Glaas et al. [30]; Hawker [36]; Johannsdottir et al. [43]; Keskitalo et al. [48]; Linnerooth-Bayer et al. [50]; Mills [53]; Mills [54]; Müller-Fürstenberger and Schumacher [56]; Paudel [63]; Stahel [79]; Thirawat et al. [85]; Ward et al. [95]; Wilkins [97]; Woods and Urwin [99]
(9) Accountability and reporting	Gatzert and Reichel [28]; Ho et al. [38]; Pierro and Desai [66]; Scholtens [76]; Sethi [77]

The statistics reported in Table 2 highlight an important academic research gap, particularly in the categories sales and marketing and claims management where the number of publications is low. However, our statistics also show that the environmental issue has received the most attention over the past two decades, particularly regarding risk management and underwriting. Social and governance factors are less studied, although they get some attention in relation with investment management.

### Statistics on practitioner publications along the value chain categories

**Table 4:** Number of practitioner publications per category and ESG factor

	Number of records			
Category	Overall	E	S	G
(1) Company strategy	5	3	3	2
(2) Product and service development	9	8	2	2
(3) Sales and marketing	1	1	0	0
(4) Risk management and underwriting	15	14	5	6
(5) Claims management	8	7	3	2
(6) Investment management	11	8	6	3
(7) Clients, suppliers and investors	6	5	5	4
(8) Government and regulatory bodies	12	11	4	5
(9) Accountability and reporting	8	8	4	5

The column “Overall” refers to the total number of practitioner publications in a given category. The columns “E”, “S” and “G” stand for the environmental, social and governance factors. A publication may relate to several categories and a category to several ESG factors

In this section, we first summarize the 23 practitioner studies along the nine categories of the insurance value chain (see Sect. 3.3). In analogy to Table 2 for the academic publications, we report in Table 4 the number of records in practitioner studies in each category and ESG factor. We observe that, as with the academic publications, the risk management and underwriting (4) category is the most discussed in practitioner publications. In the sales and marketing (3) category, we found only one study dealing with the environmental topic. However, we can also observe that categories that have been less researched in academia, such as claims management (5), clients, suppliers and investors (7), and accountability and reporting (9) are of great interest to practitioners. In addition, we note that environmental risks are the most studied by practitioners when compared to social and governance risks. We provide more details on the selected practitioner publications in Table 8 in the Appendix.

As mentioned in Sect. 3 , we have categorized the practitioner contributions into four distinct types. For the United Nations group, we have identified the UNEP Finance Initiative PSI, the global framework for insurance-specific treatment of ESG issues, with four publications. For the group of regulators and standard setters, we have considered the main body in Europe for insurance companies and pension funds, the European Insurance and Occupational Pensions Authority (EIOPA) with three publications, an international body, the International Association of Insurance Supervisors (IAIS) with one publication, and the insurance-specific network dealing with sustainability issues, the Sustainable Insurance Forum (SIF) with two publications. We excluded national regulatory bodies whose ESG related publications typically take the form of directives. The “insurance” group encompasses records from the main public organizations, private companies (e.g., Swiss Re, Allianz, Munich Re, AXA, Zurich, Aviva, Generali) and think tanks. After review, we included the Geneva Association (GA) with four publications, the Chief Risk Officers Forum (CRO Forum), and Swiss Re with two publications in our corpus. In the actuarial group, we have considered the International Actuarial Association (IAA) with four publications and the Actuarial Association of Europe (AAE) with one publication. In Table 5, we provide an overview of the publications and the classification along the nine value chain categories.

**Table 5:** Classification of the practitioner studies (by type) along the value chain categories

Organization		Reference and *title*	(1)	(2)	(3)	(4)	(5)	(6)	(7)	(8)	(9)
United Nations	UNEP PSI	UNEP Finance Initiative [87]: *Managing ESG Risks in Non-Life Insurance Business*				$\checkmark$			$\checkmark$		$\checkmark$
		UNEP Finance Initiative [90]: *Insuring the Net-Zero Transition: Evolving Thinking and Practices*				$\checkmark$			$\checkmark$	$\checkmark$	
		UNEP Finance Initiative [91]: *Managing ESG Risks in Life and Health Insurance Business*				$\checkmark$			$\checkmark$		$\checkmark$
		UNEP Finance Initiative [92]: *New Risks, New opportunities: Harnessing EPLI for a Suitable Economy*		$\checkmark$		$\checkmark$	$\checkmark$			$\checkmark$	
Regulators	EIOPA	EIOPA [23]: *Report on Non-Life Underwriting and Pricing in Light of Climate Change*		$\checkmark$	$\checkmark$	$\checkmark$	$\checkmark$				
		EIOPA [24]: *European Insurers’ Exposure to Physical Climate Change Risk*				$\checkmark$	$\checkmark$				
		EIOPA [25]: *Guidance on the Integration of Sustainability Preferences in the Suitability Assessment under the IDD*						$\checkmark$	$\checkmark$		
	IAIS	Bourtembourg et al. [13]: *The Impact of Climate Change on the Financial Stability of the Insurance Sector*						$\checkmark$			
	SIF	Baral [8]: *Nature-Related Risks in the Global Insurance Sector*		$\checkmark$		$\checkmark$		$\checkmark$		$\checkmark$	$\checkmark$
		IAIS [39]: *Issues Paper on the Implementation of the TCFD Recommendations*	$\checkmark$							$\checkmark$	$\checkmark$
Insurance	GA	Golnaraghi and Geneva Association [33]: *Insurance Industry Perspectives on Regulatory Approaches to Climate Risk Assessment*								$\checkmark$	
		Golnaraghi and Geneva Association [34]: *Anchoring Climate Change Risk Assessment in Core Business Decisions in Insurance*	$\checkmark$	$\checkmark$		$\checkmark$				$\checkmark$	
		Golnaraghi and Mellot [35]: *Nature and the Insurance Industry: Taking Action Towards a Nature-Positive Economy*		$\checkmark$		$\checkmark$		$\checkmark$		$\checkmark$	
		Schanz [74]: *The Role of Insurance in Promoting Social Sustainability*	$\checkmark$			$\checkmark$		$\checkmark$	$\checkmark$		
	CRO Forum	CRO Forum [16]: *Imagine All the People: Demographics and Social Change from an Insurance Perspective*	$\checkmark$	$\checkmark$			$\checkmark$	$\checkmark$			
		CRO Forum [17]: *Mind the Sustainability Gap: Integrating Sustainability into Insurance Risk Management*	$\checkmark$			$\checkmark$		$\checkmark$		$\checkmark$	$\checkmark$
	Swiss Re	Swiss Re [83]: *Remote Sensing Innovation: Progressing Sustainability Goals and Expanding Insurability*		$\checkmark$		$\checkmark$	$\checkmark$				
		Swiss Re [84]: *Reshaping the Social Contract: the Role of Insurance in Reducing Income Inequality*						$\checkmark$		$\checkmark$	
Actuarial	IAA	Crugnola-Humbert et al. [18]: *Climate-Related Disclosures and Risk Management: Standards and Leading Practices*				$\checkmark$				$\checkmark$	$\checkmark$
		Meins et al. [51]: *Pension Fund ESG Risk Disclosures: Developing Global Practice*						$\checkmark$		$\checkmark$	$\checkmark$
		Musulin et al. [57]: *Climate-Related Scenarios Applied to Insurers and Other Financial Institutions*		$\checkmark$		$\checkmark$	$\checkmark$	$\checkmark$			
		Wason et al. [96]: *Importance of Climate-Related Risks for Actuaries*		$\checkmark$		$\checkmark$	$\checkmark$	$\checkmark$			$\checkmark$
	AAE	Armengol Vivas et al. [4]: *Sustainability Issues and Reputational Risk for Insurance Companies and Pension Funds*					$\checkmark$		$\checkmark$	$\checkmark$	

The column “Organization” includes the organization’s type (i.e., United Nations, Regulators, Insurance, and Actuarial) and name. The abbreviations are as follows: *AAE* Actuarial Association of Europe, *CRO Forum* Chief Risk Officers Forum, *EIOPA* European Insurance and Occupational Pensions Authority, *GA* The Geneva Association, *IAA* International Actuarial Association, *IAIS* International Association of Insurance Supervisors, *SIF* Sustainable Insurance Forum, *UNEP PSI* United Nations Environment Programme Principles for Sustainable Insurance. In the column “Reference and *title*”, the abbreviation EPLI stands for Environmental Pollution Liability Insurance. The columns labeled (1) to (9) correspond to the categories introduced in Sect. 3.3, see also Fig. 4

## Results and discussion

In this section, we review the retrieved publications and discuss their contents. In Sect. 4.1, we first describe the relevance to sustainability of each framework category (Fig. 4) and put the existing research, including both academic and practitioner publications, in context. In Sect. 4.2, we summarize the main results of the extant literature in terms of issues and related actions (see Table 6), and, in the light of the findings, we discuss the current state of the insurance industry with regards to sustainability issues.

### Review of the literature

**Company strategy.** As a first step towards becoming a sustainable company, the strategy must be well defined and include sustainability at all levels of the organization. With the goal of identifying and monitoring ESG issues in business operations, insurers need consistent corporate strategy at the board and executive levels. The strategy must integrate sustainability into all business areas and the corporate culture, and determine appropriate quantitative indicators to measure progress [81].

Research related to sustainable strategies is found in the area of corporate sustainability, which refers to companies addressing sustainability issues, including economic, environmental, and social factors (see, e.g., [68] for a systematic literature review). Corporate governance is important for the strategy to develop well-structured frameworks [44, 99]. For example, in the case of socially responsible pension funds and with the demand for sustainable development, institutional shareholders care more about social and environmental values, which they transfer to corporate governance [2].

In addition, the literature emphasizes the importance of using green technologies [9, 50] as the main strategy for climate adaptation. Environmental sustainability and climate change actions need to be integrated into the insurers’ core business [45]. Altarhouni et al. [3] insist that all actors of the insurance sector, including insurers, reinsurers and pension funds, should develop strategies to reduce environmental degradation by investing in clean energy sources. Furthermore, corporate social responsibility (CSR) has been identified as a key practice for the strategy of insurance companies and pension funds [38, 78]. The insurance industry can play a pioneering role in CSR. Insurance companies typically have incorporated social and ethical issues into their business activities, but not environmental issues, so that many insurers do not realize their full potential for a more sustainable industry [76]. The article by Ho et al. [38] describes that for Taiwanese insurance companies, managerial practices are the most significant dimension of CSR, with corporate strategies and commitments the most important criteria. However, we note that the strategy area is not well documented in our final corpus, particularly regarding social and governance factors.

**Product and service development.** In our framework, we consider the development of products and services as the first operations activity of an insurer. Our literature review highlights that it is necessary to distinguish between the products and services offered by insurance companies in developing countries and in developed countries. For example, in developing countries, climate-related microinsurance products are more in demand by policyholders than most of such products in the traditional market [54]. In addition, there is also a growing demand for weather-related and nature-aligned products in developed countries. These insurance products can also reduce underwriting losses, stimulate the growth of insurance assets, and help restore damaged natural capital [8]. Such propositions include crop parametric products for drought risks based on a soil moisture index and property (flood) parametric products based on an excess rainfall index [83]. Regarding social issues, the world is experiencing an aging population, especially in Europe. Given the pressure on public budgets, it is likely that society and governments will be very receptive to more creative and affordable insurance and pension propositions. There are already examples of interactions between governments, individuals, and private companies, such as insurers and asset managers working together on the definition of the pan-European personal pension product (PEPP), a voluntary personal pension scheme, complementary to state-based and occupational pensions, that offers EU citizens a new option to save for retirement [16].

Therefore, the development of sustainable products and services provides opportunities and challenges for the core insurance business [58]. For instance, one challenge is to design and price products that consider climate risks and stakeholders. For insurance companies, this may mean raising premiums or excluding coverage in areas at elevated risk for climate-related events such as floods or bushfires. On the other hand, there is an opportunity to develop products that align policyholder interests with behaviors that lead to better climate outcomes. This could be achieved by introducing incentives that eliminate or control risks, low-carbon annuity products, or providing capital for initiatives that address climate risks [96].

One of the main goals of sustainable insurance is to develop products and services that reduce risks and have a positive impact on ESG issues. Possible actions suggested by the PSI include offering microinsurance to developing countries where access to insurance is limited and supporting programs on risk, insurance, and ESG issues [86]. Some insurance companies have developed “green” insurance products and features. Examples include the coverage for electric and hydrogen vehicles, discounts for low- and zero-emission vehicles, and for policyholders who use public transportation, repair rather than replace, and coverage for sharing mobility [81]. Other examples contributing to environmental sustainability include electric cars and renewable energy solutions [45]. The insurance industry has developed products such as environmental pollution liability insurance in response to the rise of liabilities it has faced over time [92]. Insurance companies that find the best solutions with innovative and sustainable products and services will lead the insurance markets in the future.

**Sales and marketing.** The least researched category with merely three retrieved academic publications and one practitioner study in our literature review is sales and marketing. Nevertheless, the associated operational processes are key for sales promotion, advertising, channel relationships and distribution. Indeed, an appropriate marketing strategy can make customers aware of sustainability issues and provide them with the best option for their expectations. To meet the requirements of a sustainable company, PSI suggests, first, educating sales staff about ESG issues and, second, integrating key messages responsibly into campaigns and social media [86]. The sales and marketing team must understand and explain the (sustainability) benefits and costs of each product offered by the company. For example, promoting an insurer’s efforts to mitigate weather disasters is an important tool in a marketing strategy to strengthen customer loyalty [49, 95].

Currently, insurance companies are already actively promoting extreme weather risk management to raise awareness of how the industry is responding to the impacts of climate change [95]. However, there is a potential risk to insurers’ reputations if false advertising, known as “greenwashing”, is used as in marketing campaigns [29]. In addition, the lack of common definitions and standards for measuring the contribution of products and services to climate change mitigation and adaptation may increase the risk of greenwashing [23]. One suggestion to avoid the image of using greenwashing as marketing is to apply standards, best practice and established measures (see, e.g., the Science-Based Target initiative, SBTi, [73], and [81]).

**Risk management and underwriting.** Actuaries play an active role in risk management and underwriting. In this category, we focus on liability and underwriting in the context of insurance companies’ and pension funds’ risk management. The extant literature focuses primarily on environmental issues, highlighting the interest in developing measures to manage, measure and mitigate the increase in natural disasters.

In their role as risk managers, insurance companies provide expertise in catastrophe risk modeling, risk assessment, and preventive measures [32]. An alarming figure that illustrates the importance of underwriting is that the economic cost of weather damage alone could exceed USD 1 trillion by 2040 worldwide [21]. Thus, underwriters are facing huge challenges, mainly because catastrophe models are not well calibrated, premiums are too low, risk exposures are extremely high, and the climate protection gap is growing. However, increasing insurance coverage alone is not enough to close the protection gap, as the increasing frequency and severity of certain natural events could make some risks uninsurable. Measures that can be implemented include proactive mitigation initiatives for buildings, localization of risks, and optimized levels of insurance coverage [26]. Another survey study on flood damages in the United Kingdom reports insurance losses from floods of GBP 1.7 billion in 2007 [7]. To respond to these challenges, the academic literature has studied several actions. For instance, Begum et al. [9], discusses how green technology strategies can help to reduce the impact of natural disasters in Malaysia. In addition, Botzen et al. [12] suggests that increasing crop insurance policies is a business opportunity to cover drought losses in the Netherlands. Toward best practices, Mills [54] indicates that appropriate risk management in businesses requires a reassessment of existing risk management tools in response to environmental issues. On a broader level, Glaas et al. [30] shows the importance of government involvement for insurer stability and risk prevention measures.

Insurers’ risk management requires immediate action to address the consequences of climate change, at the global and local levels due to the increase in extreme natural disasters and the associated risk of increasing insurance losses [37, 61, 63, 72]. In addition, the increase of natural catastrophes does not only come with an increased risk in terms of frequency and severity, but the frequency of these events is more unpredictable over time [65]. Moreover, the consideration of comprehensive ESG risk assessments beyond the pure environmental aspects is critical when the developing risk management and underwriting. One of the keys to sustainable development in insurance risk management is technological innovation [19]. By developing and providing knowledge to mitigate losses through risk engineering and insuring sustainable technologies, the sector can play an important role [79]. In addition, especially in the case of climate change, the use of specific financial instruments such as cat bonds and catastrophe risk swaps are important tools for disaster risk management [85].

With respect to sustainability risks, risk management and underwriting are a top business priority and must be addressed by actuaries. According to the PSI, appropriate processes to identify and assess ESG issues should be incorporated into the portfolio, the modeling, and all analyses done within the company [86]. The top priority in risk management is to invest in the mitigation of losses. Mitigation and prevention of disasters can range from reducing risk exposure to creating institutions for better response, such as land use and emergency planning [50]. An interesting five-step roadmap for risk management, aimed primarily at small and medium-sized insurance companies, has been proposed by Stricker et al. [81], and consists of integrating sustainability into risk assessments, reviews of risk objectives, setting tolerance levels, risk monitoring and reporting, and external environment monitoring.

**Claims management.** Given the share of an insurer’s costs related to claims, claims management is fundamental to insurers and presents potential opportunities. However, claims management has not been largely studied from a sustainability perspective: only two publications appear in our literature review. The article by Sato and Seki [72] discusses the case of Japan and emphasizes that insurance companies must have a well-structured claims assessment process that promotes precautionary measures within the company. This is critical to minimize losses from natural disasters and to improve claims payments. Two examples for moving towards a greener industry in claims management are the use of image recognition technologies in claims assessments, such as photos of local repair companies, and the creation of a greenhouse gas emissions inventory for all operations and processes in claims management [81].

To achieve sustainable insurance, a comprehensive claims management strategy including stakeholders, namely, the customers, must be on the agenda of insurance companies. A critical part of an insurer’s value chain is managing the total cost of risk from claims. Root cause analysis, repairing a loss, and improving claims management strategies are examples of better practices. In recent years, the number of climate-related claims has increased [96]. In terms of physical risk, worker’s compensation claims are a relevant example since climate can affect mortality and morbidity risks at work. It takes longer for claims to develop, and new sources of risk may emerge. Reserving methods may be affected by changes in claims payment patterns that are difficult to determine from historical data [57]. One of the main concerns for insurers is damage to their reputation in terms of how well they can insure sustainability risks, keep them affordable, and provide good service after catastrophic losses. One solution is smart claims payments to “build back better” after a disaster. This approach could increase the resilience of society and better align the interests of insurers and customers [4]. Another solution is the use of remote sensing, which simplifies existing processes such as claims management. For example, remote sensing-based flood detection can provide access to timely insights for claims developments. Furthermore, the use of technologies from remote sensing satellites can help assess the risk of subsidence, by measuring soil movement, and identify claims for property insurance [83].

**Investment management.** Insurance companies and pension funds are among of the largest institutional investors in the global economy. Therefore, investment management is a relevant lever for addressing sustainability issues. Importance also comes with the long-term investment horizon. Our literature review shows that insurers’ investment practices are well researched. Moreover, there is an undeniable growing interest in considering ESG investment practices in the insurance industry [2]. So-called “sustainable investing” is of great importance for the sector and investors’ interest has increased in recent years [28].

Several studies highlight the benefits of ESG investments with a positive impact on investment decisions, such as the National green technology policy in Malaysia [9]. Similarly, appropriate solutions addressing climate change provide profitable investment opportunities [80]. Nevertheless, climate change also comes with higher risks, for example, regarding real estate investments. For this reason, life insurance companies, which mainly own long-term assets, are more affected by climate change than property insurance companies [69]. Among the investment strategies, ESG integration and impact investing are the two most frequently mentioned in recent years [28]. Five main mitigation measures are proposed in pension fund sustainability reports: divestment, direct engagement, carbon footprint calculation, investing in green options, and participating in climate-related coalitions. Long-term sustainable investments are likely to be beneficial and successful, especially in the area of retirement insurance [60]. As a result, some companies have already incorporated exclusion criteria in their portfolio strategies to respond to environmental risks [15]. However, the use of these measures has been counterproductive in some pension funds in taming the fossil fuel sector. For instance, some pension funds practice industry-wide divestment, such as from tobacco and nuclear weapons, although they rarely target the fossil fuel sector. Other pension funds practice conditional divestment, where they only divest from a company as a last resort if a set of criteria is not met [70]. In addition, the incorporation of ESG practices into the investment management of pension funds and insurance companies, essentially socially responsible and environmental investing, is increasingly required by the market and should be considered a priority in decision making [54]. On the opportunity side, a proposed solution to climate change, especially for large insurers, is to fund client projects that improve resilience [80].

One of the most important guidelines related to sustainability in investments are the *principles for responsible investment* [88], whose defined goal is to achieve sustainable global finance by incorporating ESG factors into investment portfolios. In addition, standards have been developed, particularly on the social factor such as, *socially responsible investing*, which includes social factors as well as areas like community investment and shareholder advocacy [31]. Investors have used ESG information mainly through ratings, which help transform ESG data into investment products for decision making. Nonetheless, ESG practices are gaining traction in the insurance sector, with further development of ratings required [11]. Existing data providers in the market include Bloomberg, Morningstar, Thompson Reuters, MSCI, and Sustainalytics, which tend to focus on financial institutions. They consider some of the key criteria within the three ESG factors. For example, environmental factors include carbon emissions, pollution, and natural resource use; social factors include health and diversity issues, human rights, privacy, and community engagement. Under the corporate governance factor, we find, among other things, corporate ethics, board independence and shareholder rights. Many of the largest pension funds are actively working to improve their ESG practices, however there is still room for improvement. Overall, integrating ESG issues into investment management is critical to achieving better long-term investment returns and addressing climate risks [51].

**Clients, suppliers and investors.** External stakeholders include clients, suppliers and investors. Principle 2 of the PSI specifically addresses these crucial players in the insurance industry. Our literature review highlights that long-term relationships with policyholders and customers are one of the most important mechanisms for insurers to pursue an integrated sustainability strategy [71]. The practice of CSR engagement by insurers has recently had a positive impact on customer behavior [49]. Regarding the environmental factor, incentives for customers with ESG commitment lead them to consider greener options in the insurance industry [97]. For example, insurance companies in Massachusetts have offered discounts of 10% to customers who complete a free six-hour course on weatherization^3^ and home repairs [52].

Customers are also concerned about the social factor. On the one hand, for example, pension funds get involved by, e.g., informing pension fund members about where their funds are invested and whether they are invested considering ESG issues. In non-life insurance, policyholders expect that coverage will continue to be available and affordable, and that claims will be settled at an elevated level after a catastrophe event. However, sustainability risks can disrupt this and negatively impact clients and potentially lead to reputational risks for insurers [4]. On the other hand, insurers should promote corporate commitment to sustainability issues through marketing strategies to attract more policyholders and raise awareness in the global community. Thereby, it is important to act responsibly on all ESG issues. Based on guidance from the PSI, a number of actions have been proposed, such as starting a dialogue with policyholders and suppliers about the benefits of ESG management and the company’s sustainability expectations and encouraging policyholders and suppliers to use relevant ESG disclosures [86]. Insurers have to work together with their clients, suppliers, and investors to contribute to a sustainable business.

**Government and regulatory bodies.** Climate change is an example showcasing the need for insurance companies, governments, and society to develop sustainable solutions together [36, 42]. Regulation can boost the sustainability agenda and change the way business is done. Regulating the integration of ESG factors can help to reduce risks and better manage sustainability (cf. the current efforts by the EIOPA to explicitly integrate sustainability in the solvency regulation, see [22]). Thereby, regulatory bodies improve the monitoring of sustainability issues. In addition, governments, along with insurers and society, are important players in promoting ESG issues, especially in areas where more capital is needed, as in the case of extreme weather events [54]. Governments can influence private insurance companies in adapting to sustainability with a longer-term perspective. For example, the Norwegian government has initiated an approach to public-private compensation of insurers for costly extreme events, called the Norwegian natural perils pool [30]. The Swiss natural perils pool (“Elementarschaden-Pool”) also intends to optimize risk diversification and risk exposure among the participating insurers covering 90% of the natural perils market [82]. In Europe, however, only a quarter of climate-related and extreme event losses have been insured over the past 40 years, revealing a climate protection gap [26]. Other factors in which governments can be involved include providing a water management solution and establishing monitoring systems for extreme natural events [85]. Another example of government involvement can be found in the United Kingdom, where the government works with private insurers to provide flood insurance [95].

Two important actions proposed by the PSI include dialogue with intergovernmental and nongovernmental institutions to promote sustainable development, and support for policy, regulatory, and legal bodies that enable risk mitigation and better management of ESG issues [86]. Policy options that the state can use include motivating technology developers to behave prudently by creating a liability regime, encouraging innovation ideas by creating non-liability regimes and effectively allocating risk to third parties, promoting insurance in the risk market by setting liability limits, and promoting social equity by making insurance mandatory [19].

**Accountability and reporting.** The literature shows that it is of utmost importance to publicly disclose the actions on the relevant challenges in terms of ESG factors [38]. Currently, insurance companies and pension funds, particularly in Europe, have begun to report annually on their progress and actions related to environmental and sustainability issues. Since 2015, numerous standards and regulatory measures have been developed for sustainability, sustainable finance, and climate-related disclosures. An analysis by the UNEP has found that around 25% of these measures address disclosure of ESG factors, sustainability, and climate risks [39]. One of the most influential organizations on climate issues is the Task Force on Climate-related Financial Disclosures (TCFD), which recommends climate reporting based on four main pillars (governance, strategy, risk management, and metrics and targets, see [18]). In sustainability reporting, one of the main guidelines used in practice are those of the Global reporting initiative, whose standards aim to strengthen sustainability and transparency in the insurance market. Another practice increasingly used in the insurance industry is CSR reporting. The European Commission adopted the Corporate Sustainability Reporting Directive (CSRD) in April 2021, which replaces the Non-Financial Reporting Directive (NFRD) and broadens the scope of reporting requirements. The CSRD mandates companies to furnish information that enables an understanding of the impact of sustainability-related issues on the company and its effects on people and the environment [17, 74].

Potential actions suggested by the PSI include monitoring and measuring the company’s progress in addressing ESG issues, participating in relevant reporting frameworks, and engaging in dialogue with regulators and rating agencies to gain a mutual understanding of the value of disclosure. Reporting has become increasingly important in recent years. Annual reporting on progress and actions on ESG issues is the means to inform policymakers and society on how the company is responding to sustainability issues [86]. Therefore, information disclosure, reporting, accountability, and transparency are key to achieve sustainable insurance and must be a priority for the industry today.

### Discussion of sustainability issues and related actions

To complement the literature review presented in Sect. 4.1, we propose a summary of the main findings in Table 6: we lay out the main sustainability issues and some potential related actions per category that appear in the final corpus of literature.

**Table 6:** Sustainability issues and potential related actions per category

Category	Main sustainability issues	Potential related actions
(1) Company strategy	No well-defined corporate strategy for sustainability	Integrate ESG issues into all business processes
	Absence of sustainable governance framework	Adopt indicators and measure progress in implementing sustainability
	Lack of appropriate quantitative indicators	Make green technologies the main strategy for climate adaptation
(2) Product and service development	Growing demand for “green” products and services	Develop products that contribute to environmental sustainability (e.g., weather-related, low-carbon, and renewable energy solutions)
	Limited access to insurance in developing countries	Coverage for electric and hydrogen vehicles, and sharing mobility
	Design and price climate-related products taking into account stakeholders	Support of microinsurance and ESG practices in developing countries
		Introduce incentives that eliminate risks, and provide capital for climate initiatives
(3) Sales and marketing	Greenwashing	Apply existing standards (science-based targets) and practices
	Education and knowledge transfer	Integrate key messages responsibly into campaigns and social media
(4) Risk management and underwriting	High economic costs due to weather damages	Incorporate ESG factors into modelling and research
	Increased frequency and severity of catastrophe events	Use of global risk pooling strategies (e.g., catastrophe risk swaps)
	Unpredictable frequency of natural disasters	Provide knowledge to mitigate losses with technological innovation
	Growing climate protection gap	Establish tolerance levels for catastrophe coverage
		Implement proactive mitigation initiatives for buildings
(5) Claims management	Lack of ESG factors integration in claims management	Embed ESG issues into claims services
	Reputation damage for maintaining affordable prices and good service after a catastrophic loss	Smart claims payment to “build back better” after a disaster
		Use of image recognition technologies for claims investigations
		Use remote sensing to simplify and accelerate existing processes
(6) Investment management	Real estate is exposed to greater climate risks	Implement climate policies (e.g., divestment, direct engagement, carbon footprint calculation, “green”/alternative investments)
	Lack of appropriate financial tools for NatCat risks	Use of alternative instruments to cover losses (e.g., cat bonds and swaps)
	Increasing climate change leads to rising financial risks	Fund customer projects that improve resilience
(7) Clients, suppliers and investors	Expectation of affordable coverage after a disaster	Engage to corporate social responsibility practices
	Awareness and education of intermediaries	Incentivize clients with ESG commitments
	Dialogue with external stakeholders	Encourage stakeholders to use relevant ESG disclosures
(8) Government and regulatory bodies	Lack of regulatory frameworks including ESG factors	Build legal frameworks for risk mitigation of ESG issues
		Dialogue with government and external bodies to gain support
(9) Accountability and reporting	Lack of accountability and transparent reporting at the micro level	Monitor and measure the progress in addressing ESG issues
		Participate in relevant reporting frameworks
		Engage in dialogue with regulators and rating agencies

As sustainable insurance has become a key objective, it is undeniable that insurance companies and pension funds must act responsibly to respond to the ESG challenges of our time. Building socio-economic resilience is at the core of the insurance business. Companies need (to develop) well-structured risk management and adaptation strategies [32]. They also need to understand the exclusion and inclusion criteria for ESG investments and manage them in a sustainable manner. In addition, our literature review has revealed that specific attention must be paid to insurance activities such as sales and marketing, claims management, and clients, suppliers and investors, as there is a lack of research in these areas. When considering the publications, we also observe that most of the articles focus on developed countries and issues from climate change. We also find some specific actions and proposals that the insurers have incorporated into their strategy and operations. Academic research shows that the issues of sustainability and insurance are growing and are important to both researchers and practitioners. Indeed, there is no doubt that numerous challenges arise when developing adaptation.

We have seen a lot of attention paid to sustainability in financial markets. Insurance companies are conducting ESG assessments, largely due to pressure from the society, their customers and regulators. At the time of writing, several initiatives and principles have become available and insurance companies must achieve a net-zero greenhouse gas emissions commitment by 2050 [89]. However, understanding the definitions and the lack of data, regulations, standards, and best practices make it difficult for insurers to achieve the goal.

Small and medium-sized insurance companies need to put sustainability on their agenda and start the transition by implementing actions and measures that will help the entire sector become a sustainable business. Furthermore, not only small and mid-sized insurers, but all actors need to consider the complex sustainability journey when outlining the path to implementation. A comprehensive roadmap for green insurance along the value chain, particularly for small and medium-sized insurers, is proposed by Stricker et al. [81], which identifies five key steps in each area of the insurer value chain. The steps include insuring green attributes in the product and service development category, integrating sustainability into risk management and underwriting, and inventorying greenhouse gas emissions in the claims management area.

Our study on the current state of literature reveals number of elements hindering the sustainable development in insurance. First, we note that the insurance sector depends on external factors beyond its control, such as regulatory frameworks and shareholder decisions. In addition, there are few data sets, metrics, and theories to model ESG risks. Furthermore, the insurance industry needs a detailed roadmap and process for implementing sustainability that is not yet well defined. For example, while the measures proposed in the PSI provide general guidance on the four principles, they say little about how they should be implemented in practice, particularly with regard to the first principle for company strategy, claims management and sales and marketing. Another limitation relates to the different ESG assessments. For example, different investors approach ESG factors differently; some companies have already fully integrated ESG factors, while others are just beginning to implement them. Moreover, ESG assessments are valued differently by different investors and agencies [98]. Finally, the definition of sustainability must be reliable and endure over time. As a prominent example of that uncertainty, we note that the scope of green energy in Europe has recently changed, and, under the EU Taxonomy, nuclear energy has been classified as green [40].

## Conclusion

In this work, we present the results of a systematic literature review on sustainability in insurance companies and pension funds. Our results provide an overview of the main sustainability issues and related actions along the key insurance activities. Guided by the PSI and the insurance value chain, the review and the summary findings are structured along nine categories relating to the company strategy, six core operations (product and service development, sales and marketing, risk management and underwriting, claims management, investment management), and clients, suppliers and investors, government and regulatory bodies, as well as accountability and reporting. Although academic research has seen important developments in recent years, we have identified gaps, for example in the areas of claims management and marketing. Throughout the publications, we observe that the academic community acknowledges that acting on sustainability issues is not only a stake in the future, but a real challenge in the present for the insurance sector.

In terms of ESG factors, the environmental factor is the most prevalent across all categories in our literature. On the liability side, insurance companies are directly affected by climate change risks. For the risk management and underwriting operations, many academics call for more metrics and models to manage ESG risks. On the other hand, on the asset side, insurers and pension funds can actively influence the future through their institutional investments and related engagements. We observe that both insurance companies and pension funds focus mainly on environmental and social factors in their sustainable investments. Despite their interest in governance issues and although it is a fundamental part of corporate strategy, the fact that governance is not insurance-specific and more difficult to measure could be the reason there are only few publications addressing this issue in the insurance literature. In addition, in our literature review, we note that the governance factor is usually not discussed alone, but together with one or two factors, i.e., environmental, or social factors. This is particularly the case in the practitioner studies. Overall, we find that more detailed sustainability roadmaps need to be developed and guidelines require more thorough integration. The latter thoughts should also include areas such as claims management and sales and marketing. Accountability and reporting, an area that has seen significant developments recently, is of great interest to practitioners, and there is no doubt that academic publications on this topic will also increase in the coming years.

While the present contribution provides a review of the existing literature, further research should be considered as an extension. First, many of the current models in risk management and underwriting do not yet incorporate ESG factors, leaving room for future research and requiring engagement from actuaries. Second, specifically for the environmental factor, further studies can be conducted on the impact of climate change-related risks on pricing, underwriting, risk management, and solvency. Finally, more research is also required on the impact and implementation of specific social and governance factors. This will be particularly relevant since their modeling and measurement in practice is more difficult.

## Data Availability

Not applicable.

## Appendix

See Tables 7 and 8.

**Table 7:** Synopsis of the academic research articles identified

Reference	Region	Meth.	Key contents and main results	(1)	(2)	(3)	(4)	(5)	(6)	(7)	(8)	(9)	E	S	G
Alda [1]	UK	DP	Social responsible (SR) pension funds are influential institutional shareholders in investee firms	$\checkmark$					$\checkmark$				$\checkmark$	$\checkmark$	$\checkmark$
			SR pension funds impact on many of the ESG indicators studied from 2002 to 2018												
			Firms with larger pension-fund shareholding are positively influence the ESG firm performance												
Alda [2]	UK	DP	Analysis of considering ESG factors in conventional pension funds investing						$\checkmark$				$\checkmark$	$\checkmark$	$\checkmark$
			Conventional and social responsible investing funds have similar ESG concerns												
			Limited availability period and lack of ESG scores for conventional funds												
Altarhouni et al. [3]	TR	DP	Exploration of the role of insurance market development on environmental degradation	$\checkmark$									$\checkmark$		
			Positive influence of insurance development on environment and carbon emissions												
			Policymakers should make strategies to ensure the sustainable development of insurance												
Autenne et al. [5]	EU	DP	Sustainability of pension systems cannot ignore the importance of the financial dimension						$\checkmark$				$\checkmark$	$\checkmark$	$\checkmark$
			Socially responsible and environmentally sensitive investments must be considered												
			Ensuring maximum long-term returns must be considered together the sustainability notion												
Ball et al. [7]	UK	SV	Question on how best to manage flood risk, linking defenses to the insurability of properties				$\checkmark$				$\checkmark$		$\checkmark$		
			Resilience in flood-risk management implies that total prevention of flood damage is not possible												
			Insuring high flood-risk areas must be compatible with sustainable flood-risk management policies												
Begum et al. [9]	MY	DP	Overview of Malaysia’s national green technology policy and disaster risk reduction	$\checkmark$	$\checkmark$		$\checkmark$		$\checkmark$				$\checkmark$		
			Green technologies can offer risk management benefits for insurance industry												
			New paradigm based on prevention of losses and participation in green technology marketplaces												
Benali and Feki [10]	US	DP	Natural disasters encourage insurers to take measures and stabilize technical performance								$\checkmark$		$\checkmark$		
			Volume of capital and premium to surplus ratio have a positive impact on profitability												
			Loss ratio, unexpected frequency and blockbuster negatively effect insurers’ profitability												
Botzen et al. [12]	NL	DP	Overview of the consequences of climate change for insurers and new business opportunities				$\checkmark$						$\checkmark$		
			Economic losses caused by extreme weather events are expected to increase												
			Adequate climate change projections must be included in risk management												
Brogi et al. [14]	US	DP	Interaction between financial ratios and ESG awareness of 107 insurers								$\checkmark$		$\checkmark$	$\checkmark$	$\checkmark$
			Size, profitability and solvency are critical for ESG awareness and policy implementation												
			Proposed model allows to unfold ESG practices in the insurance industry												
Chiaramonte et al. [15]	US	DP	As insurers target long-term objectives, they benefit the most from sustainable engagements						$\checkmark$		$\checkmark$		$\checkmark$	$\checkmark$	$\checkmark$
			ESG scores improve insurers’ stability, driven primarily by environmental and social factors												
			Life insurers benefit more than non-life insurers from corporate social responsibility												
Dahlström et al. [19]	EU	CS	Study focuses on interplay between the risk insurability, innovation and sustainable development		$\checkmark$		$\checkmark$				$\checkmark$		$\checkmark$	$\checkmark$	$\checkmark$
			Manage novel risk and promote sustainability using risk management mechanisms from insurers												
			Disconnection between the investment and underwriting is an obstacle to act on sustainability												
Dlugolecki [21]	UK	DP	Economic cost of weather damage could reach over USD 1 trillion in a single year by 2040				$\checkmark$		$\checkmark$				$\checkmark$		
			Adaptation, disaster management and sustainable development require public-private cooperation												
			In underwriting, current catastrophe models are wrongly calibrated and premiums are too low												
Garayeta et al. [27]	EU, US, CN, AU, BR, ZA	DP	Determination of the degree of sustainability of solvency systems in insurance markets				$\checkmark$				$\checkmark$		$\checkmark$	$\checkmark$	$\checkmark$
			Changes in solvency systems are mainly related to governance factors (AU, CN and ZA rank first)												
			Regulators should develop scenarios for long-term sustainability of insurance companies												
Gatzert and Reichel [28]	EU, US	LR	European insurers report more extensively on sustainable investment practices than US						$\checkmark$			$\checkmark$	$\checkmark$	$\checkmark$	$\checkmark$
			References are made to ESG criteria and sustainable development goals												
			Top investment strategies mentioned are ESG integration and impact investing												
Glaas et al. [30]	DK, NO, SE	LR	Political factors influence approaches to managing climate change more than market factors				$\checkmark$				$\checkmark$		$\checkmark$		
			Public-private collaboration in climate change risk prevention measures is suggested												
			Government interventions are vital for establishing long-term planning horizons in insurers												
Hawker [36]	AU	CS	Less predictable climate reduces the insurers’ capacity to price and spread weather-related risk								$\checkmark$		$\checkmark$		
			General insurance is a necessary community product and needs to be available and affordable												
			Better understanding of long-term opportunities and risks is needed												
Herweijer et al. [37]	UK	DP	In the short term, climate change affects underwriting: risk quantification must be adapted				$\checkmark$		$\checkmark$				$\checkmark$		
			New approaches include forward-looking views on risk not purely grounded in historical experience												
			In the longer term, insufficient adaptation where risk is rising threatens insurability itself												
Ho et al. [38]	TW	SV	Managerial practices are the most important dimension of corporate social responsibility in insurers	$\checkmark$								$\checkmark$	$\checkmark$	$\checkmark$	$\checkmark$
			Reference indicators for the insurers to promote sustainable development												
			Information disclosure, corporate strategies and legal compliance are criteria for sustainability												
Johannsdottir [42]	NC	DP	Business strategies must integrate climate change measures (benchmarking, reporting, disclosure)	$\checkmark$									$\checkmark$		
			Exploration of the Geneva Association framework for climate change actions												
			(In-)activeness relates to company size and outside drivers like market and social drivers												
Johannsdottir et al. [43]	NC	CS	Non-life insurance sector is under-performing in fulfilling climate commitments		$\checkmark$		$\checkmark$				$\checkmark$		$\checkmark$		
			The smaller insurers are not participating into climate-related actions (mitigation, adaptation)												
			Illustration of examples of climate actions that are taken by Nordic insurers												
Johannsdottir et al. [45]	NC	CS	Environmental issues can be addressed in strategy, operations, products, and investments	$\checkmark$	$\checkmark$				$\checkmark$				$\checkmark$		
			Employee acceptance of change increases with increased environmental awareness												
			Strategic maps highlight environmental sustainability strategy implementation												
Johannsdottir and McInerney [44]	NC	CS	Framework to implement sustainability goals into culture, core business, strategy, and structure	$\checkmark$									$\checkmark$		
			Five phases: commitment, configuration, core business, communication, continuous improvement												
			Case study and interviews involving leading non-life insurers operating in the Nordic region												
Keskitalo et al. [48]	UK, DE, NL	DP	Study on the adaption to climate change and the renegotiation of responsibility for flood risk		$\checkmark$						$\checkmark$		$\checkmark$		
			Compulsory natural hazard insurance option in Germany is neither financially nor legally feasible												
			In the Netherlands, the development of short-term private flood insurance is suggested												
Lee et al. [49]	TW	LR	Corporate social responsibility activities enhance brand image, reputation and policyholder loyalty			$\checkmark$			$\checkmark$	$\checkmark$				$\checkmark$	
			Policyholders are more satisfied with insurers that are more socially responsible												
			Non-life insurance should promote CSR to reduce social and environmental risks												
Linnerooth-Bayer et al. [50]	DC	DC	Examination of insurance offering affordable economic security to vulnerable communities	$\checkmark$			$\checkmark$				$\checkmark$		$\checkmark$		
			Insurance mechanisms to be included in climate adaptation regimes against weather disasters												
			High costs of catastrophe cover can be not affordable for low income clients												
Mills [52]	n.a.	DC	Energy-efficient and renewable energy technologies that offer loss-prevention benefits		$\checkmark$		$\checkmark$		$\checkmark$	$\checkmark$			$\checkmark$		
			Presentation of the business case for insurer involvement in the sustainable energy sector												
			European insurance are less active in the promotion of energy efficiency and renewable energy												
Mills [53]	n.a.	DC	Climate change affects affordability and availability, shifting burden to governments/individuals				$\checkmark$				$\checkmark$		$\checkmark$		
			Risk-management products and loss-prevention technologies are business opportunities												
			Insurance can develop microinsurance to fund disaster preparedness in developing countries												
Mills [54]	US	SV	Recognition of Enterprise Risk Management as a framework for assessing climate risks	$\checkmark$	$\checkmark$		$\checkmark$		$\checkmark$	$\checkmark$	$\checkmark$		$\checkmark$		
			Insurers must adapt climate modeling and role of regulators must be clarified												
			Climate microinsurance has more clients than most climate products in the traditional market												
Mills [55]	n.a.	DP	One in eight insurance companies in US have a formal strategy for climate-risk disclosure				$\checkmark$						$\checkmark$		
			Insurers are exposed to governance risks and those taken by their customers												
			Little climate-related innovation has occurred in life and health, maritime and aviation industries												
Müller-Fürstenberger and Schumacher[56]	n.a.	DP	The more transparent the insurance, the more sustainable the economic and environmental system				$\checkmark$				$\checkmark$		$\checkmark$		
			A decentralized economy will not invest enough in emissions reduction without policy interventions												
			Precautionary beliefs about the frequency of extreme events lead to greater sustainability												
Nogueira et al. [58]	BR	SV	Today’s corporate social responsibility assessment procedures do not use ESG risk factors		$\checkmark$		$\checkmark$						$\checkmark$	$\checkmark$	$\checkmark$
			Proposal of an integrative model to understand how underwriting influences management												
			Environmental and governance constructs are discriminants; company size relates to progress												
Owadally et al. [60]	EU, US	DP	Long-term investing along ESG principles is likely to be successful in securing retirement income						$\checkmark$				$\checkmark$	$\checkmark$	$\checkmark$
			Index investing with ESG score-based screening is a key sustainable strategy for pension planning												
			Transactions costs and investment management fees are excluded in the study (passive investment)												
Pagano et al. [61]	n.a.	LR	Exploration of adaptation measures against climate change by insurance companies				$\checkmark$						$\checkmark$		
			Insurers’ tools include methodologies and models able developing an acceptable risk forecast												
			Description of specific cases, including, e.g., risk transfer tools to natural disaster												
Paudel [63]	DE, UK, ES, CH	DP	Comparison of private, public, and public-private insurance systems for extreme events				$\checkmark$				$\checkmark$		$\checkmark$		
			Mandatory insurance participation in extreme events is needed for a high rate of market penetration												
			Government needs to provide a state guarantee for uncertain catastrophic risks												
Phelan [65]	n.a.	DP	Insurance may cease being a tool for climate risks in the absence of effective climate mitigation				$\checkmark$						$\checkmark$		
			Climate change threatens the increase in weather event frequency and the climate risk probabilities												
			Deep and rapid cuts in emissions are the only way to avoid uninsurable climate risks												
Pierro and Desai [66]	ET, MW, IN	DP	Assessment of micro- and macro-level weather insurance schemes as tools for social protection	$\checkmark$								$\checkmark$	$\checkmark$		
			Macro schemes to index-based insurance seem to be suitable for low-probability weather risks												
			Macroinsurance schemes have more potential than microinsurance to protect the most vulnerable												
Qing and Liang [69]	CN	DP	Dual role of insurance: reduce impact of climate change and adapt through products and services		$\checkmark$		$\checkmark$		$\checkmark$				$\checkmark$		
			Insurers in China can save energy in office building lighting, cooling, and vehicle usage												
			Regulators must allow insurance to charge premiums that reflect climate change threats												
Rempel and Gupta [70]	OECD	DP	Actions for pension funds: divest, engage, calculate carbon footprint and investing in alternatives		$\checkmark$				$\checkmark$				$\checkmark$		
			Pension funds are not yet fully committed to leaving fossil fuels underground												
			It is important that pension funds use coalitions to maximize their leverage over fossil firms												
Risi [71]	CH	SV	Frameworks to estimate sustainability risks hamper socially responsible investing practices						$\checkmark$	$\checkmark$				$\checkmark$	
			Insurers’ risk can prevent active disclosure of ESG issues and adoption of better practices												
			The calculation of short-term ESG risk encourages the adoption of reactive SRI practices												
Sato and Seki [72]	JP	DP	Beyond education, there are two approaches to address climate change: mitigation and adaptation				$\checkmark$	$\checkmark$					$\checkmark$		
			Insurance should reduce CO$_2$ emissions drastically and develop products for climate change issues												
			Japanese insurers will use risk management expertise to achieve a climate-resilient society												
Schiller and Crugnola-Humbert [75]	n.a.	DP	Actuaries can support the creation of new accounting approaches and disclosure frameworks				$\checkmark$						$\checkmark$		
			New forward-looking techniques will be needed for assessing sustainability risks												
			The inclusion of carbon emission pricing may be a valuation approach used by actuaries												
Scholtens [76]	EU, NA, JP	DP	Investigation of corporate social responsibility ratings of insurance companies	$\checkmark$								$\checkmark$		$\checkmark$	
			Social aspects are better integrated into insurers’ activities than environmental issues												
			European and Japanese insurers perform better than North American insurers												
Sethi [77]	US	DP	Pension funds can improve the quality of socially responsible corporate conduct	$\checkmark$			$\checkmark$		$\checkmark$	$\checkmark$		$\checkmark$		$\checkmark$	$\checkmark$
			Large corporations need to become active agents for social change												
			Corporate participation in social policy formulation is necessary												
Sievänen et al. [78]	EU	SV	Analysis of the interaction of responsible investing and financial investing in pension funds	$\checkmark$					$\checkmark$				$\checkmark$	$\checkmark$	$\checkmark$
			Pension funds with strong financial focus are very likely to include responsibility into their strategy												
			There is a balance between finance and responsibility in pension who integrate sustainability												
Stahel [79]	n.a.	DP	Insurance can provide expertise for loss prevention and sustainable investments via risk engineering		$\checkmark$		$\checkmark$		$\checkmark$		$\checkmark$		$\checkmark$		
			Insurability of technological innovations would increase the competitiveness of low-risk technologies												
			Insurance is a key player on the investment and underwriting side in public-private partnerships												
Stechemesser et al. [80]	n.a.	SV	Adaptation to climate change arises from climate knowledge, operational flexibility and integration						$\checkmark$				$\checkmark$		
			Adaptation generates profit for insurers relating to better corporate financial performance												
			Positive relationship between ROA and climate change knowledge and awareness												
Stricker et al. [81]	n.a.	DP	Complex interdependencies, non-linear effects and externalities hamper the pricing of ESG risks	$\checkmark$	$\checkmark$	$\checkmark$	$\checkmark$	$\checkmark$					$\checkmark$		
			Sustainability must be embedded into the corporate culture and along the insurance value chain												
			Insurers need to analyze sustainability exposure, revise objectives and define transition actions												
Thirawat et al. [85]	TH	DP	Catastrophe bonds are a useful instrument for disaster risk management	$\checkmark$	$\checkmark$		$\checkmark$		$\checkmark$		$\checkmark$		$\checkmark$		
			Collaboration with the World Bank would be beneficial for managing catastrophe risks												
			Alternative global risk pooling strategies include catastrophe risk swaps												
Ward et al. [95]	US	DP	Insurers can incentivize clients to invest in measures that mitigate the impacts of extreme weather			$\checkmark$					$\checkmark$		$\checkmark$		
			Coordination with governments and policymakers can help to provide better coverage for risks												
			Insurers can promote adaptation to mitigate the impact of climate change in society												
Wilkins [97]	AU	DP	Insurance sector needs multiple level actions to deal with climate change adequately		$\checkmark$		$\checkmark$			$\checkmark$	$\checkmark$		$\checkmark$	$\checkmark$	
			Multi-level approach includes engaging governments, educating and incentivizing												
			Insurance must address the challenges of climate change while balancing the needs of stakeholders												
Woods and Urwin [99]	UK, US	DP	Proposal of a governance framework to implement sustainable investing in pension funds	$\checkmark$					$\checkmark$		$\checkmark$				$\checkmark$
			Framework includes ESG factors, ownership, economic and legal challenges of the future												
			Investment beliefs must be reconsidered to adapt governance practices to sustainable investing												

In the column “Region”, the abbreviations are as follows: *AF* Afghanistan, *AU* Australia, *BR* Brazil, *CH* Switzerland, *CN* China, *DC* Developing countries, *DE* Germany, *DK* Denmark, *ES* Spain, *ET* Ethiopia, *EU* European Union member states, *IN* India, *JP* Japan, *MW* Malawi, *MY* Malaysia, *NA* North America, *NC* Nordic countries, *NL* Netherlands, *NO* Norway, *SE* Sweden, *TR* Turkey, *TW* Taiwan, *UK* United Kingdom, *US* United States, *ZA* South Africa. The abbreviation “n.a.” stands for not applicable relating to studies without empirical analyses. The column “Meth.” informs on the methods used: *CS* case study, *DP* discussion paper, *LR* literature review, *SV* survey. In the column “Key contents and main results”, the abbreviation ROA stands for return on assets. The columns labeled (1) to (9) correspond to the categories introduced in Sect. 3.3, see also Fig. 4. The last three columns (E, S, G) stand for the environmental, social and governance factors, respectively

**Table 8:** Synopsis of the practitioners studies articles identified

Reference	Org.	Type	Key contents and main results	(1)	(2)	(3)	(4)	(5)	(6)	(7)	(8)	(9)	E	S	G
[4]	AAE	Act.	Identification and assessment of reputational risks, including developing protection gaps					$\checkmark$		$\checkmark$	$\checkmark$		$\checkmark$	$\checkmark$	$\checkmark$
			Reputational risk can be an opportunity and ESG reporting requirements can be used positively												
			Actuaries contribute to strong risk culture and with long-term perspectives												
[8]	SIF	Reg.	Nature loss can manifest into physical and transition financial risks for the insurance sector		$\checkmark$		$\checkmark$		$\checkmark$		$\checkmark$	$\checkmark$	$\checkmark$		
			As natural events increase, associated financial risks could increase in severity and frequency												
			Insurance takes a wider view of nature-related risks and develops measurement tools												
[13]	IAIS	Reg.	First quantitative global study on the climate change impact on the insurance sector						$\checkmark$				$\checkmark$		
			Climate risks bring challenges, such as the lack of a globally consistent framework												
			More than 35% of insurers’ investment assets could be considered climate relevant												
[16]	CRO Forum	Ins.	Demographic changes and the growth of the elderly population have important consequences	$\checkmark$	$\checkmark$			$\checkmark$	$\checkmark$					$\checkmark$	
			Technological changes in many dimensions lead to an intensive use of automation												
			European insurers need to consider the impact of increasing urbanization changes												
[17]	CRO Forum	Ins.	Presentation of a set of guidelines for integrating sustainability into insurers’ risk management	$\checkmark$			$\checkmark$		$\checkmark$		$\checkmark$	$\checkmark$	$\checkmark$	$\checkmark$	$\checkmark$
			Coverage of the nature of sustainability, including dual materiality and dynamic materiality												
			Examples of applying risk management to sustainability in underwriting, investment and others												
[18]	IAA	Act.	Climate-related disclosures provide information on the potential impacts of climate change				$\checkmark$				$\checkmark$	$\checkmark$	$\checkmark$		$\checkmark$
			TCFD is one of the most influential standards for sustainability and climate disclosures												
			Many countries have adopted specific regulations for companies to disclose climate information												
[23]	EIOPA	Reg.	Short-term feature of non-life contracts is the argument for not including climate risks in pricing		$\checkmark$	$\checkmark$	$\checkmark$	$\checkmark$					$\checkmark$		
			Insurers can play a key role in climate protection gap, contributing to mitigation and adaptation												
			Introduction of the concept of impact underwriting in the development of products												
[24]	EIOPA	Reg.	Assessment of insurers’ exposure to physical risks from the financial stability perspective				$\checkmark$	$\checkmark$					$\checkmark$		
			Insurers may experience an increase in frequency and severity of natural catastrophe claims												
			All property insurance lines are expected to be affected by the physical risks of climate change												
[25]	EIOPA	Reg.	Consultation on guidelines for incorporating clients’ sustainability preferences under the IDD						$\checkmark$	$\checkmark$			$\checkmark$	$\checkmark$	$\checkmark$
			Simple document is needed to understand the integration of customers’ preferences												
			Promotes better understanding of the new rules coming into effect												
[33]	GA	Ins.	Usage of scenario analysis for climate risk assessment is a recommendation of the TCFD								$\checkmark$		$\checkmark$		
			Climate risk analysis requires a forward-looking approach and a focus on concrete objectives												
			Development of methods and tools for assessing climate risks is still in progress												
[34]	GA	Ins.	Guidance for re/insurers to implement climate change risk assessment and scenarios analysis	$\checkmark$	$\checkmark$		$\checkmark$				$\checkmark$		$\checkmark$		$\checkmark$
			Ongoing development of global baseline standards for sustainability reporting												
			Upcoming implementation of legal requirements for climate change risk disclosure												
[35]	GA	Ins.	Sustainability of natural capital is essential for socio-economic development and prosperity		$\checkmark$		$\checkmark$		$\checkmark$		$\checkmark$		$\checkmark$		
			WEF 2022 report lists nature degradation and biodiversity loss as major long-term risks												
			Nature-based solutions increase resilience to physical climate risks												
[39]	SIF	Reg.	TCFD has helped inform market and supervisory practice related to climate risk disclosures	$\checkmark$							$\checkmark$	$\checkmark$	$\checkmark$		
			Understanding of TCFD remains low and few insurers have plans to implement them												
			Supervisors may consider broader climate issues, such as the impact on insurance pricing												
[51]	IAA	Act.	ESG disclosure is limited but increasing rapidly as legislation is implemented						$\checkmark$		$\checkmark$	$\checkmark$	$\checkmark$	$\checkmark$	$\checkmark$
			Discussion of ESG disclosures in relation to pension funds and their investments												
			Coverage of financial and reputational risks to pension funds and their long-term performance												
[57]	IAA	Act.	Scenario analysis as core component of climate-related risk assessment		$\checkmark$		$\checkmark$	$\checkmark$	$\checkmark$				$\checkmark$		
			Guidance on challenges faced in implementing climate-related scenario analysis												
			Use of case studies to identify relevant information to assess risks and opportunities of a firm												
[74]	GA	Ins.	Insurers and public struggle to understand social or “S” factors in investment and business decisions	$\checkmark$			$\checkmark$		$\checkmark$	$\checkmark$				$\checkmark$	
			Insurance is recognized as socially beneficial and contributes positively to social sustainability												
			Insurers need to pay more attention to the “S” dimension, providing benefits and avoiding risks												
[83]	Swiss Re	Ins.	Applications of geodata and earth observation data are rapidly increasing in various industries		$\checkmark$		$\checkmark$	$\checkmark$					$\checkmark$	$\checkmark$	$\checkmark$
			Advancements of machine-learning techniques are key to detailed risk and loss information												
			Remote sensing will enable new markets and risk pools and simplify existing processes												
[84]	Swiss Re	Ins.	Income inequality in countries is negative for social cohesion, economic growth and insurance						$\checkmark$		$\checkmark$			$\checkmark$	
			Economic shocks affect lower-income households, and sustained inequality has negative effects												
			Insurers can work with public sector to reach underserved communities												
[87]	UNEP PSI	UN	One of the first ESG guides for global non-life insurance business				$\checkmark$			$\checkmark$		$\checkmark$	$\checkmark$	$\checkmark$	$\checkmark$
			Realization of over 50 interviews to understand the lack of ESG policies in the industry												
			Guidance for development approaches to assessing ESG risks in non-life insurance												
[90]	UNEP PSI	UN	Outline of NZIA’s works to develop the first worldwide standard for measuring insurance emissions				$\checkmark$			$\checkmark$	$\checkmark$		$\checkmark$		
			Changes to the atmosphere and biosphere are leading to more frequent and intense events												
			NZIA works on challenge to achieve net-zero emissions within the underwriting portfolios												
[91]	UNEP PSI	UN	Guide providing assistance to life and health insurers in linking ESG and business impact				$\checkmark$			$\checkmark$		$\checkmark$	$\checkmark$	$\checkmark$	$\checkmark$
			Development of the practice guide supports the aims of the four PSI												
			Support tool to help insurers, particularly those with limited ESG knowledge												
[92]	UNEP PSI	UN	Role of insurance in leading the mobilization of capital for sustainable investments		$\checkmark$		$\checkmark$	$\checkmark$			$\checkmark$		$\checkmark$		
			Better alignment of assets and liabilities in insurers is needed in terms of sustainability												
			Emergence of insurance policies in response to specific policy and regulatory requirements												
[96]	IAA	Act.	Actuarial organizations to contribute to climate risks through research and development		$\checkmark$		$\checkmark$	$\checkmark$	$\checkmark$			$\checkmark$	$\checkmark$		
			Actuaries need to learn and adapt to improve their ability to identify climate risks												
			Inability to assess relevant risks can be seen as a failure of management and directors												

In the column “Org.” we display the organization’s name, and the abbreviations are as follows: *AAE* Actuarial Association of Europe, *CRO Forum* Chief Risk Officers Forum, *EIOPA* European Insurance and Occupational Pensions Authority, *GA* The Geneva Association, *IAA* International Actuarial Association, *IAIS* International Association of Insurance Supervisors, *SIF* Sustainable Insurance Forum, *UNEP PSI* United Nations Environment Programme Principles for Sustainable Insurance. The column “Type” describes the association or group type using the following abbreviations: *Act.* Actuarial, *Ins.* Insurance, *Reg.* Regulators, *UN* United Nations. Other abbreviations used are as follows: *FSD* Financial Stability Board, *IDD* Insurance Distribution Directive, *NZIA* Net-Zero Insurance Alliance, *TCFD* Task Force on Climate related Financial Disclosures, *WEF* World Economic Forum. The columns labeled (1) to (9) correspond to the categories introduced in Sect. 3.3, see also Fig. 4. The last three columns (E, S, G) stand for the environmental, social and governance factors, respectively

## Footnotes

1 The exact query is as follows: AB=((“insur*" OR “pension*" OR “actuar*") AND (“sustainab*" OR “esg") AND (“environment*" OR “soci*" OR “govern*" OR “climate change")).

2 In forward tracking we consider the publications that have cited a given record; in backward tracking we analyze the references cited in a given record.

3 Courses on weatherization provide basic knowledge of building science related to airflow, heat flow and moisture flow, combustion safety and testing. They are designed in accordance with guidelines specified by the U.S. Department of Energy, see https://www.greentrainingusa.com/doe-weatherization-installer-1-and-2-training.html.
